# Environmental spreading of clinically relevant carbapenem-resistant gram-negative bacilli: the occurrence of *bla*_KPC-or-NDM_ strains relates to local hospital activities

**DOI:** 10.1186/s12866-021-02400-1

**Published:** 2022-01-04

**Authors:** Alex Leite Pereira, Pâmela Maria de Oliveira, Célio Faria-Junior, Everton Giovanni Alves, Glaura Regina de Castro e Caldo Lima, Thaís Alves da Costa Lamounier, Rodrigo Haddad, Wildo Navegantes de Araújo

**Affiliations:** 1 Campus of Ceilândia, University of Brasília. Centro Metropolitano, Conjunto A, Ceilândia Sul, Brasília, DF CEP: 72220-275 Brazil; 2 Central Laboratory for Public Health (LACEN-DF), SGAN 601, Asa Norte, Brasília, DF CEP: 70830-010 Brazil

**Keywords:** Carbapenem resistance, Sewage treatment, Surveillance

## Abstract

**Background:**

Aquatic matrices impacted by sewage may shelter carbapenem-resistant (CR) Gram-negative bacilli (GNB) harboring resistance genes of public health concern. In this study, sewage treatment plants (STPs) servicing well-defined catchment areas were surveyed for the presence of CR-GNB bearing carbapenemase genes (*bla*_KPC_ or *bla*_NDM_).

**Results:**

A total of 325 CR-GNB were recovered from raw (RS) and treated (TS) sewage samples as well as from water body spots upstream (UW) and downstream (DW) from STPs. *Klebsiella-Enterobacter* (KE) group amounted to 116 isolates (35.7%). CR-KE isolates were recovered from TS, DW (35.7%) and RS samples (44.2%) (*p* = 0.001); but not from UW samples. KE isolates represented 65.8% of all *bla*_KPC_ or *bla*_NDM_ positive strains. The frequency of *bla*_KPC-or-NDM_ strains was positively associated with the occurrence of district hospitals located near STPs, as well as with the number of hospitalizations and of sewer connections serviced by the STPs. *bla*_KPC-or-NDM_ strains were recovered from ST samples in 7 out of 14 STPs, including four tertiary-level STPs; and from 6 out of 13 DW spots whose RS samples also had *bla*_KPC-or-NDM_ strains.

**Conclusions:**

Clinically relevant GNB bearing *bla*_KPC-or-NDM_ resist sewage treatments and spread into environmental aquatic matrices mainly from STPs impacted by hospital activities.

**Supplementary Information:**

The online version contains supplementary material available at 10.1186/s12866-021-02400-1.

## Background

Carbapenems are last-resort antibiotics for treating infections caused by multidrug-resistant (MDR) Gram-negative bacilli (GNB). The spread of carbapenem-resistant (CR) strains has concerned physicians and hospital health managers, becoming an unprecedented threat to public and environmental health [1]. In hospital environments, CR-GNB surveillance was urged as soon as the worldwide emergence of *K. pneumoniae* strains harboring resistance gene *bla*_KPC_ was confirmed [2]. *bla*_KPC_-positive strains (hereinafter simply referred to as *bla*_KPC_ strains) are now detected worldwide in a variety of GNB isolates, mainly in Enterobacterales species but also in *Pseudomonas aeruginosa* and *Acinetobacter* spp. The high transmissibility of the transposon Tn*4401*, which bears *bla*_KPC_, remains the primary mechanism for the spread of carbapenem resistance among GNB strains [3, 4].

In 2010, the detection of *bla*_NDM(New Delhi Metallo beta lactamase)_-positive strains in seepage and water puddle samples exposed the need to broaden the surveillance of carbapenemase genes by incorporating environmental sampling [5]. Studies showed that *bla*_NDM_ strains were not restricted to nosocomial settings; instead, they widely emerged in the community environment of countries such as India and Pakistan [6]. Concerning Brazil, strains harboring *bla*_KPC_ or *bla*_NDM_ (*bla*_KPC-or-NDM_) emerged and were firstly detected in nosocomial environments [7–9].

Large amounts of antibiotics are discharged in sewer systems due to incomplete metabolism in humans, to the disposal of unused antibiotics and to antibiotic usage in economic activities, such as intensively managed livestock farming [10]. On account of the substantial load of bacteria and antibiotics, hospital effluents are conductive matrices for the exchange of resistance genes between pathogenic and environmental bacteria and, consequently, the selection of resistant strains [11]. Indeed, *bla*_KPC-or-NDM_ isolates of GNB have been recovered from hospital effluents worldwide [12–16], including Brazil [17, 18]. Additionally, sanitary regulations and sewage treatment processes were not conceived to face the threat of spreading antimicrobial resistance [18, 19]. In many countries, as in Brazil, hospitals are not obliged to treat their effluents before discharging them in the public sewerage [18, 20]. Moreover, even modern disinfection processes applied to sewage treatment allow the escape of CR isolates to the environment. In the United States, CR isolates were recovered from treated effluent in 42% of sewage treatment plants (STPs) using chlorination process, and in 12% of STPs using ultraviolet radiation [21]. Indeed, STPs are selective spots and reservoirs of MDR bacteria which contribute to spreading resistant strains in the environment [18, 20, 21].

The first Brazilian report on *bla*_KPC_-positive *K. pneumoniae* strains isolated from environment dated 2008 [22]. *bla*_KPC_ isolates were recovered from effluents of a STP servicing a hospital in the city of Rio de Janeiro [22]. Since then, other GNB species carrying carbapenemase genes (including *Klebisiella* spp., *Enterobacter* spp., *Kluyvera* spp., *Citrobacter* spp., *Enterobacter* spp. and *Serratia* spp.) have been recovered from coastal recreational waters [23, 24]. In Rio de Janeiro, a substantial volume of both treated and untreated sewage is continually discharged into Guanabara Bay, which in turn communicates with recreational waters of touristic beaches [23].

In Brazil, the environmental spread of *bla*_KPC-or-NDM_ isolates is predominantly reported as being a direct consequence of inappropriate sewerage infrastructure allowing the discharge of untreated sewage into the environment. We proposed a study in a different scenario. In the Brazilian capital Brasília, 85% of the sewage produced was collected in a proper sewerage and treated [25] in 14 public STPs, of which 9 carried out tertiary treatment (Table 1). Brasília (with 3 million inhabitants and an area of 5789.16 km^2^) is organized into administrative regions which differ in number of sewer connections, number of hospitals and economic profile (Table 1). Thus, in Brasília STPs serve communities located in well-defined geographical catchment areas. Additionally, hospitals in the city have reported *bla*_KPC_ strains since 2010 as well as *bla*_NDM_ strains since 2013 [7]. This study aimed to characterize the spread of *bla*_KPC-or-NDM_-positive GBN strains by way of STPs, taking into account the profile of economic activity in areas serviced by STPs, the burden on the STPs imposed by hospital services, and the level of sewage treatment achieved by STPs.

**Table 1:** Characteristics of sewage treatment plants and profile of activities in their attendance regions

	Sewage Treatment Plant (STP)			Profile of the regions serviced by the STPs					
	Flow of treated sewage (L/s)	Treatment Process^a^	Treatment accomplished	N. of sewer connections^b^	Total number of hospitals^c^	N. of district hospitals (within 3Km from STP)^c^	N. of hospitalizations (Jan-Ago 2017)^c^	Agricultural employment in the local economy (%)^d^	Areas (m^**2**^) for pig/poultry farming^e^
**1**	80	UASB+HRAP+OLF + PP	Tertiary	21,818	1	1 (1)	3010	0.6	0
**2**	41	AP + FP	Secondary	14,373	1	1 (1)	2738	11.1	0
**3**	190	UASB+AR + CLARIFIER	Tertiary	45,254	2	1 (1)	8500	1.5	179,000
**4**	767	UASB+UNITANK	Tertiary	277,076	8	2 (0)	21,624	0.2	540,000
**5**	450	BNR + AS+CP	Tertiary	63,412	6	3 (0)	13,430	0.8	0
**6**	100	UASB+HRAP+OLF	Tertiary	33,919	1	1 (1)	5607	8.3	0
**7**	155	UASB+FP + MP	Secondary	35,899	1	1 (1)	6149	9.0	0
**8**	189	UASB+AR + FP	Secondary	53,949	0	0 (0)	0	2.5	0
**9**	46	BNR + AS	Tertiary	13,079	0	0 (0)	0	0.3	0
**10**	512	UASB+FP + HRAP +PP + CP	Tertiary	74,881	1	1 (0)	5936	0.8	540,000
**11**	126	UASB+FP + HRAP+PP + CP	Tertiary	22,595	1	0 (0)	258	0.6	0
**12**	77	AC + CLARIFIER	Secondary	26,003	1	1 (1)	4561	1.3	32,000
**13**	1330	BNR + AS+CP	Tertiary	187,421	16	3 (1)	29,103	0.5	0
**14**	19	UASB+FP + MP	Secondary	4401	0	0 (0)	0	0.0	12,000

^a^*Abbreviations*: *UASB* Up-flow Anaerobic Sludge Blanket, *HRAP* High-Rate Algal Pond, *OLF* Overland Flow, *PP* Polishing Pond, *AP* Anaerobic Pond, *FP* Facultative Pond, *AR* Aerated Reactor, *BNR* Biological Nutrient Removal, *AS* Activated Sludge, *CP* Chemical Polishing, *MP* Maturation Pond

^b^Directive Plan for Waters and Sewers of the Brazilian Federal District, 2010. (Source: Companhia de Saneamento Básico do Distrito Federal, CAESB)

^c^Ministry of Health - Unified Health System (SUS) - Hospital Information System (SIH/SUS) (Accessed: May 22, 2019)

^d^Participation of agriculture in formal employment contracts by regions serviced by STP (Source: Companhia de Planejamento do Distrito Federal -CODEPLAN−/Gerência de Demografia, Estatística e Geoinformação -GEDEG. Accessed: January 30, 2019)

^e^Intensive management livestock farms operating within 3 km from the STPs (Source: Google Maps. Accessed: January 30, 2019)

## Results

Carbapenem-resistant (CR) cultures and isolates recovered from sewage and water samples.

CR cultures were produced in different proportions among the analyzed samples (supporting information Table 2). All of the 35 RS samples produced CR cultures, followed by 80% (28/35) of the TS samples, 71% (27/38) of the DW samples and 23% (7/30) of the UW samples. The proportion of CR cultures in TS and DW samples was statistically equal (*p* = 0.425); however, both proportions were higher than that found among UW samples (*p* < 0.001) (supporting information Table 2).

**Fig. 1:**
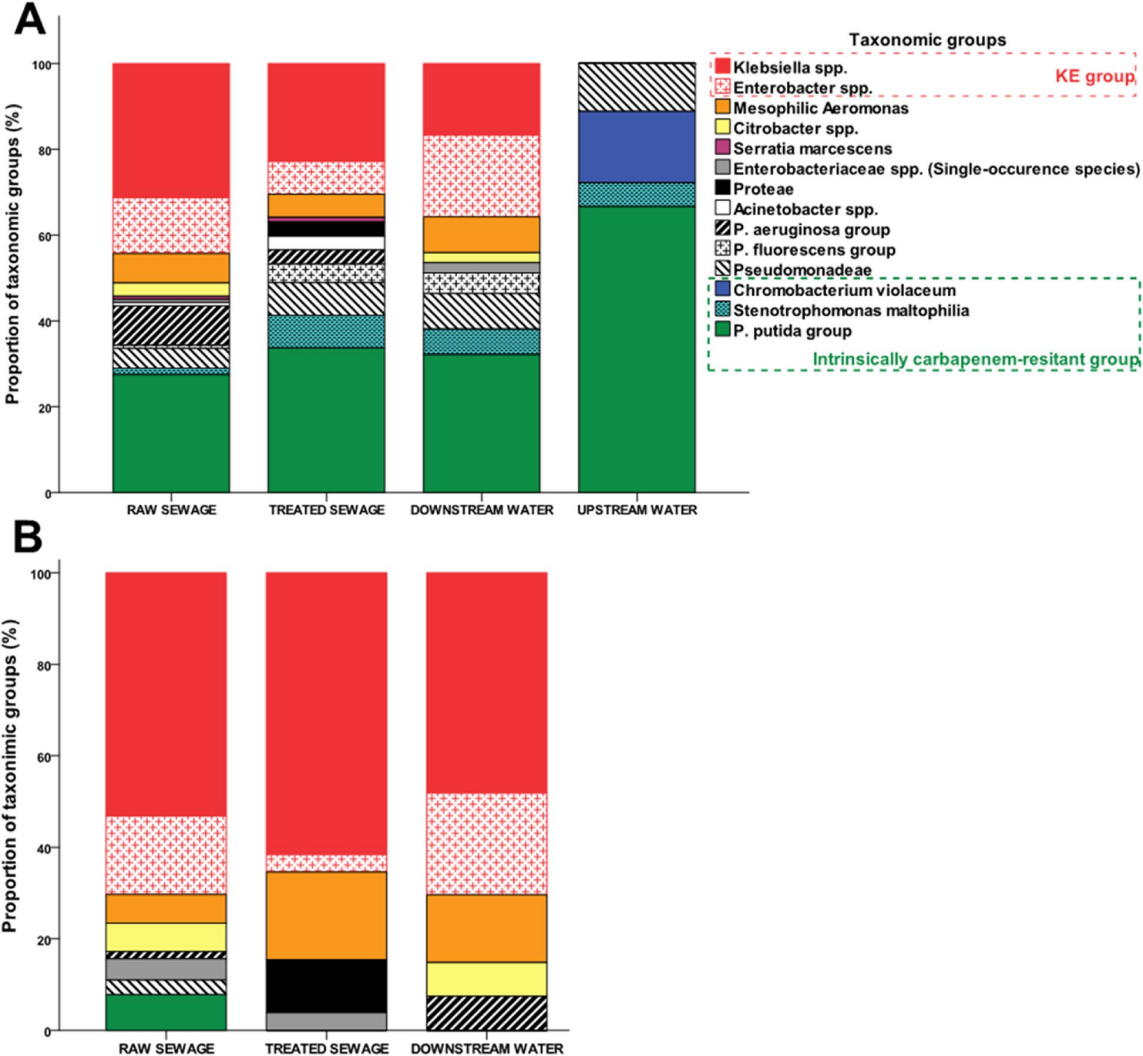
Taxonomic profile of (**A**) carbapenem-resistant and (**B**) *bla*_KPC-or-NDM_-positive isolates recovered from sewage and water samples. **A** Carbapenem-resistant isolates recovered from downstream water and sewage samples form a more diverse set of strains in comparison with the isolates from upstream water samples. Enterobacteria, especially the *Klebsiella-Enterobacter* group, are overrepresented among carbapenem-resistant isolates recovered from sewage and downstream samples. In contrast, carbapenem-resistant isolates from upstream water samples are mostly represented by intrinsically resistant, saprophyte species. **B** Strains harboring *bla*_KPC_ or *bla*_NDM_ genes (*bla*_KPC-or-NDM_) were not recovered from upstream water samples. *Klebsiella-Enterobacter* group predominates among *bla*_KPC-or-NDM_ strains recovered from downstream water and sewage samples and is followed by *bla*_KPC-or-NDM_*Aeromonas* strains

A total of 325 CR-GNB isolates were recovered and included intrinsically carbapenem-resistant species, mostly soil saprophyte species of the *Pseudomonas putida* group (32.6% - 106/325), as well as clinically relevant species, mostly isolates of the *Klebsiella-Enterobacter* (KE) group (35.7% - 116/325) (Fig. 1). It is worthy of note that mesophilic *Aeromonas* spp. accounted for 6.4% (21/325) of the CR isolates. Although isolates of *P. putida* and KE group have been equally represented, their proportions were statistically different when sorted into sample types (Fig. 1 and supporting information Table 2). Isolates of *P. putida* group were equally recovered (*p* = 0.588) from RS, TS and DW samples [respectively 27.4% (36/131), 33.6% (31/92) and 32.1% (27/84)]; however, they were recovered at a higher frequency (*p* = 0.015) from UW samples [66.6% (12/18)] (supporting information Table 2). Differently from *P. putida*, CR-KE isolates were not recovered from UW samples. Indeed, CR-KE isolates were equally recovered (*p* = 0.428) from TS and DW samples [respectively 31.5% (28/92) and 38.0% (30/84)], showing a higher recovery frequency (*p* = 0.001) from RS samples [48.0% (58/131)] (supporting information Table 2).

### Detection of carbapenemase genes in CR-GNB isolates

Carbapenemase genes were detected in 124 (39.2%) out of 316 CR-GNB isolates. The genes *bla*_KPC_, *bla*_NDM_, *bla*_IMP_ and *bla*_OXA-48_ were detected in 27.2% (*n* = 86), 10.7% (*n* = 34),1.8% (*n* = 6) and 1.2% (*n* = 4) of the tested CR-GNB, respectively. Five strains from RS samples were positive for more than one tested gene. Four isolates were positive for 2 carbapenemase genes (two *P. putida* strain with genotypes *bla*_KPC_ + bla_NMD_ and *bla*_KPC_ + *bla*_OXA-48_; one *K. pneumoniae* strain with *bla*_KPC_ + *bla*_NMD_; and one *P. aeruginosa* strain with *bla*_KPC_ + *bla*_IMP_). Furthermore, one *K. pneumoniae* strain from RS samples harbored three carbapenemase genes (*bla*_KPC_ + *bla*_NMD_ + *bla*_IMP_).

KE isolates were identified in 65.8% (77/117) of the *bla*_KPC-or-NDM_ strains (*p* < 0.0001) with the predominance of *K. pneumoniae* (53.8% - 63/117) (*p* < 0.0001), followed by *E. cloacae* (11% - 13/117) and *E. aerogenes* (3.4% - 4/117) (supporting information Table 2). Isolates of *Aeromonas* spp. carrying *bla*_KPC_ (*A. hydrophila* – *n* = 8; *A. sobria* – *n* = 4; and *A. veronii* – *n* = 1) represented 11.1% (13/117) of *bla*_KPC-or-NDM_ strains (supporting information Table 2). It is interesting to note that two uncommonly reported *bla*_KPC_-positive species of Enterobacterales species were identified: *Kluyvera ascorbata* (99.9% confidence level) and *Pantoea agglomerans* (93.3% confidence level). Lastly, the transposon Tn*4401* was detected in 97.1% (69/71) of the *bla*_KPC_ strains, including isolates of *Klebsiella* spp., *Enterobacter* spp., *Aeromonas* spp., *Citrobacter* spp., *P. putida* group., *P. aeruginosa* group, *P. agglomerans* and *K. ascorbate* (data not shown).

### Distribution of *bla*_KPC_ or *bla*_NDM_ (*bla*_KPC-or-NDM_)-positive GNB across samples

CR-GNB isolates positive for *bla*_KPC-or-NDM_ were detected at different proportions across analyzed samples (*p* < 0.001) (supporting information Table 2). *bla*_KPC-or-NDM_ strains accounted for 50.4% (64/127) of the CR-GNB isolates recovered from RS samples. The proportion of *bla*_KPC-or-NDM_ strains in TS (26/88–29.5%) and DW (27/83–32.5%) samples was statistically equal (*p* = 0.742). Conversely, *bla*_KPC-or-NDM_ strains were not detected in UW samples (supporting information Table 2). With regard to taxonomic groups, *Klebsiella-Enterobacter* group were predominant among *bla*_KPC-or-NDM_ strains recovered from RS (70.3% - 45/64), TS (65.3% - 19/27) and DW (70.3% - 17/26) samples (Fig. 1 and supporting information Table 2).

### Distribution of *bla*_KPC_ or *bla*_NDM_ (*bla*_KPC-or-NDM_)-positive GNB across STPs

**Fig. 2:**
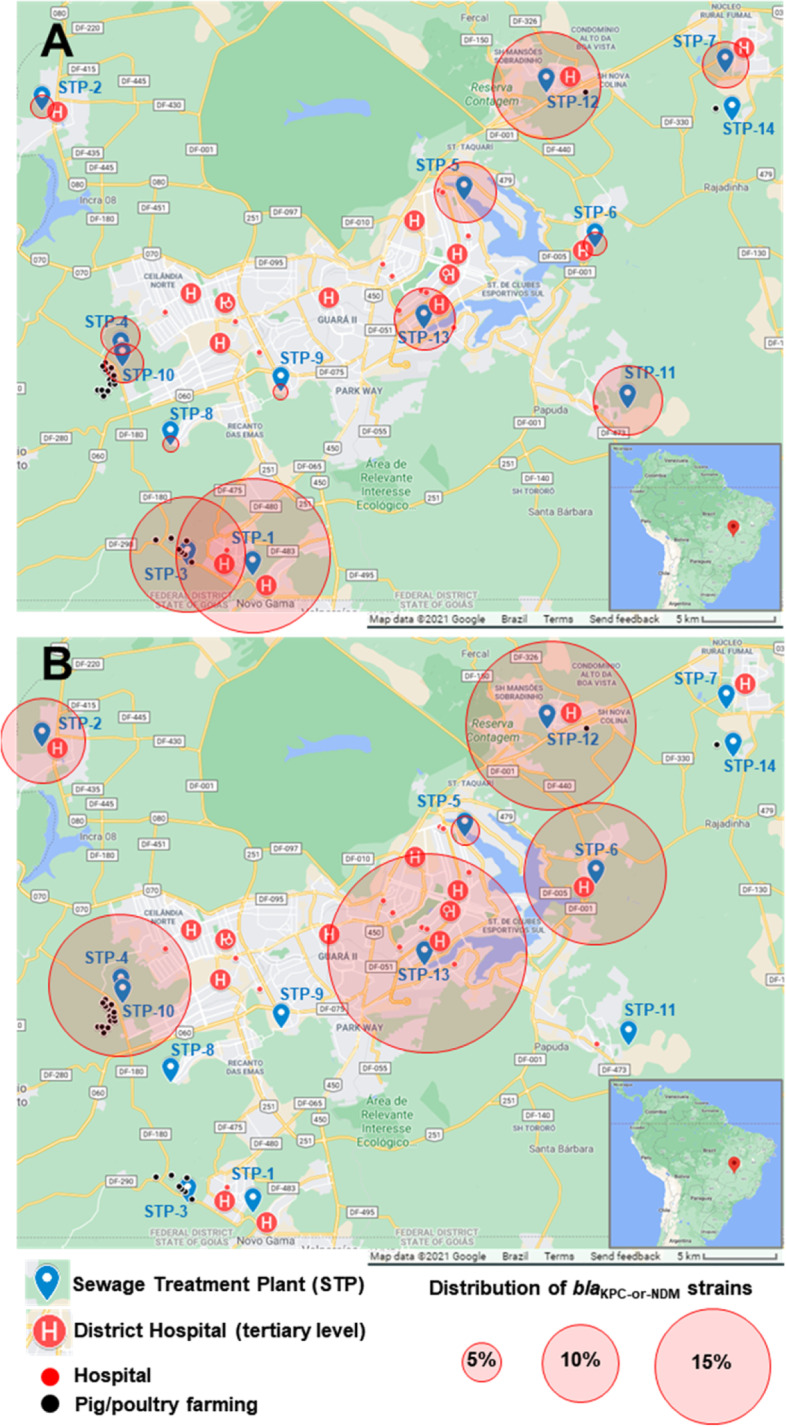
Study setting showing sewage treatment plants (STP) in Brasília, Brazil, and the distribution of *bla*_KPC-or-NDM_-positive GNB recovered from raw sewage (RS) and (B) from water bodies downstream (DW) from STPs. The insert at the bottom right displays the geographic position of the city of Brasília (red pin) located on a plateau in the central-western region of Brazil. Sewage treatment plants (blue pins), hospitals (red signals and dots) and intensive management livestock farms (black dots) are indicated. The proportional distribution among the surveyed STPs of 64 *bla*_KPC-or-NDM_ strains recovered from raw sewage (**A**) and 27 *bla*_KPC-or-NDM_ strains recovered from DW samples (**B**) is shown

In Brasília, STPs serve areas showing differences with respect to the volume of sewage produced, the number of sewer connections, the number of hospitals and hospitalizations, and the economic profile (Table 1). *bla*_KPC-or-NDM_ strains were unevenly recovered among STPs, so that 6 out of 14 STPs (STP-1, STP-3, STP-5, STP-11, STP-12, and STP-13) accounted for 75% (48/64) of the isolation of *bla*_KPC-or-NDM_ strains recovered from RS samples (Fig. 2).

**Fig. 3:**
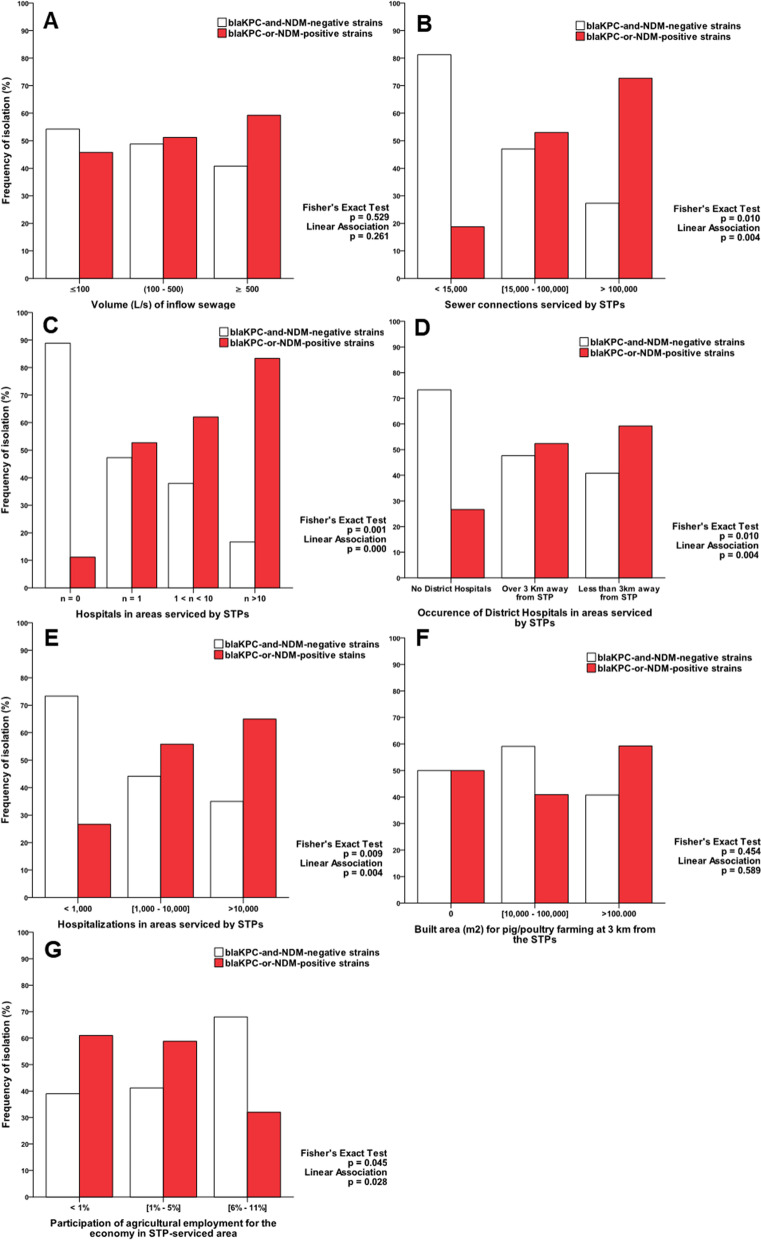
Frequency analysis of *bla*_KPC-or-NDM_ isolates recovered from STPs (raw sewage). Frequencies were distributed among categorized groups of STPs considering regional attendance profiles: **A** volume (L/s) of inflow sewage in the STPs; **B** sewer connections serviced by STPs; **C** the total number of hospitals **D** occurrence of tertiary-level district hospitals within 3 Km from the STPs; **E** the number of hospitalizations in areas serviced by STPs; **F** built area (m^2^) for pig/poultry farming within 3 Km from the STPs; and **G** participation of agricultural employment in areas serviced by STPs. Levels of significance for the distribution analysis (Fisher’s Exact Test) and for the linear relationship analysis (Cochran-Mantel-Haenszel test) are displayed in each graph

Non-parametric statistics showed that the frequency of *bla*_KPC-or-NDM_ strains increases (showing a positive association) as a function of the total number of hospitals; the number of hospitalizations; the occurrence in STP vicinities (less than 3 km away) of district hospitals; and the number of sewer connections serviced by the STPs (Fig. 3). Additionally, statistics endorsed that the total number of hospitals (mean rank of 73.8 vs. 54.0), the number of district hospitals (mean rank of 72.2 vs. 55.6) and of hospitalizations (mean rank of 71.7 vs. 56.1) ranked higher in the group of *bla*_KPC-or-NDM_ strains in comparison with *bla*_KPC-or-NDM_-negative strains (supporting information Table 3). Otherwise, the frequency of *bla*_KPC-or-NDM_ strains was statistically lower in STP areas with an increased level of agricultural employment (mean rank 52.7 vs. 67.5) (Fig. 3 and supporting information Table 3).

### Sewage treatment effect on the containment of *bla*_KPC-or-NDM_-positive strains

**Fig. 4:**
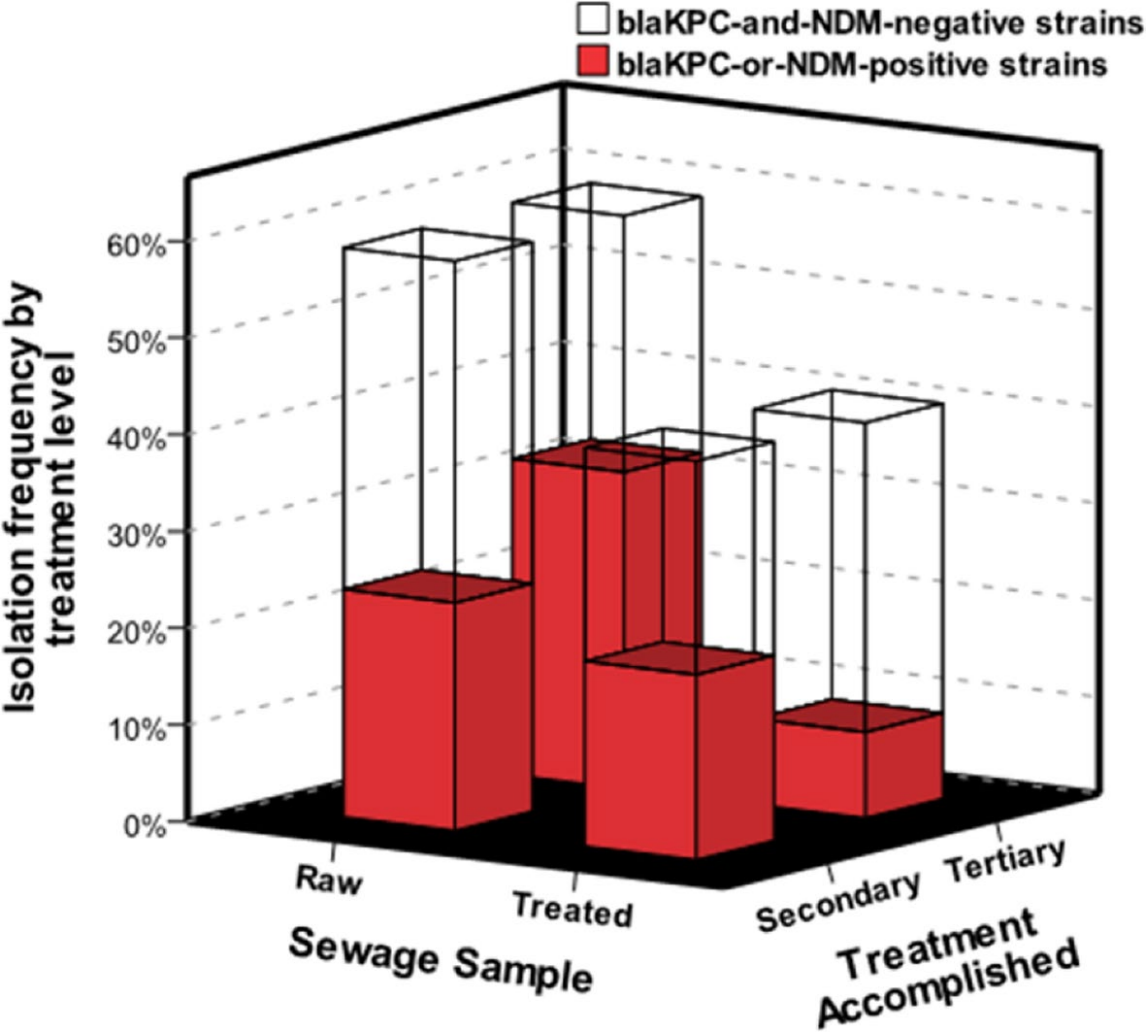
Effect of the accomplished level of sewage treatment in containing *bla*_KPC-or-NDM_ strains. Frequencies of *bla*_KPC-or-NDM_ strains recovered from raw sewage and treated sewage samples were compared considering the level of treatment (secondary or tertiary) accomplished by STPs

In order to evaluate the effectiveness of sewage treatment in reducing the spread of CR strains, the frequency of *bla*_KPC-or-NDM_ strains recovered from RS and TS samples were compared considering the treatment level (secondary or tertiary) accomplished by STPs (Fig. 4). In secondary-level STPs, *bla*_KPC-or-NDM_ strains were equally detected in RS [40% (16/40)] and TS [46.4% (13/28)] samples (*p* = 0.627). In contrast, tertiary-level STPs achieved a 33 percentage-point reduction in the frequency of *bla*_KPC-or-NDM_ strains in TS samples compared to RS samples. In tertiary-level STPs, *bla*_KPC-or-NDM_ strains were detected in respectively 55.1% (48/87) and 21.6% (13/60) of the strains recovered from RS and TS samples (*p* < 0.001).

Despite the significant reduction produced by the tertiary-level treatment, *bla*_KPC-or-NDM_ strains were still recovered from 7 out of 14 analyzed STPs, including four tertiary-level STPs (STP-4, STP-6, STP-10, STP13) and three secondary-level STPs (STP-2, STP-8, STP-12). Most of *bla*_KPC-or-NDM_ strains recovered from the TS samples belonged to *Klebsiella-Enterobacter* group (65.3% - 17/26), followed by mesophilic *Aeromonas* (19.2% - 5/26) (Fig. 1).

### *bla*_KPC-or-NDM_-positive strains evade sewage treatment, remaining viable in receiving water bodies

Environmental spread of *bla*_KPC-or-NDM_-positive strains facilitated by STPs was evaluated comparing the UW and DW samples. DW samples accounted for 23% (27/117) of all of the *bla*_KPC-or-NDM_-positive strains. In contrast, *bla*_KPC-or-NDM_-positive strains were not isolated from UW samples. *bla*_KPC-or-NDM_-positive strains were recovered from 6 out of 13 (46%) DW sites whose RS samples also had *bla*_KPC-or-NDM_ strains (Fig. 2 and supporting information Fig. 3). Additionally, five of out these six STPs had their TS samples also positive for *bla*_KPC-or-NDM_-positive strains. The leakage of *bla*_KPC-or-NDM_-positive strains was verified in four tertiary-level STPs (STP-5, STP-6, STP-4/10 and STP-13) and in two secondary-level STPs (STP-2 and STP-12) (Fig. 2 and supporting information Fig. 3).

In relation to the taxonomic groups, *Klebsiella-Enterobacter* group accounts for 70% (19/27) of *bla*_KPC-or-NDM_-positive strains recovered from DW samples, being followed by mesophilic *Aeromonas* group (14% - 4/27) (Fig. 1 and supporting information Table 2).

## Discussion

Wastewater-based epidemiology (WBE) postulates that infectious diseases and drug-related markers, including antibiotic resistance, can be monitored comprehensively through the analysis of population pooled wastewater. Additionally, WBE can provide information on the community and environmental health status as well as on the community exposure to health-threatening issues [26, 27]. Carbapenem-resistant (CR) Gram-negative bacilli (GNB) are classified as critical group for epidemiologic surveillance [1]. CR-GNB isolates recovered from diverse water matrices have been reported to shelter mobile carbapenemase genes of public health concern, such as *bla*_KPC_ and *bla*_NDM_ [28]. In this scenario, aquatic matrices have proved to be a conducive environment for the spread of the resistance to carbapenems [28].

Our survey for CR-GNB in STPs settings yielded a collection of 325 isolates. As expected, part of these isolates (124–38.2%) represented species expressing intrinsic resistance to carbapenem (*P. putida*, *S. maltophilia* and *C. violaceum*) which were frequently found in soil and water environments. These saprophytic species are not epidemiologically relevant once they rarely harbor mobile carbapenemase genes and rarely produce human infections. In contrast, 142 (43.6%) CR-GNB isolates represented intrinsically carbapenem susceptible, clinically relevant species which are frequently associated with human infections (*Klebsiella* spp., *Enterobacter* spp., *P. aeruginosa*, *Citrobacter* spp., *Serratia marcescens* and *Proteae* species).

Among 124 CR-GNB isolates harboring carbapenemase genes, 117 (94.3%) strains harbored *bla*_*KPC*_ or bla_*NDM*_, with *bla*_KPC_ strains being a majority (86–73.5%) over *bla*_NDM_ strains (34–29.0%). With regard to *bla*_KPC_-associated transposon, Tn*4401* was detected in 97% of the *bla*_KPC_ strains including Enterobacterales species, *Aeromonas* species, non-fermentative bacilli and soil saprophytes species. Therefore, our data evince the role of Tn*4401* as the foremost element for spreading *bla*_KPC_ in different species of GNB, including environmental strains [3, 4].

*Klebsiella* spp. and *Enterobacter* spp. are predominant etiologic agents of nosocomial infections [29–31]. Additionally, they account for the most of MDR isolates recovered as commensal colonizers of innate patients [29]. Patrice L Nordmann stated in mid-2010 that there would be many reasons to believe that CR *Klebsiella* spp. and *Enterobacter* spp. isolates could spread to community settings as it was extensively described for ESBL producers [32]. Indeed, CR-GNB strains are reported to be responsible for up to 29% of community-acquired infections (CAI) which frequently have *Klebsiella* spp. and *Enterobacter* spp. as aetiologic agent [33]. In this study, *bla*_KPC-or-NDM_ strains belonging to the *Klebsiella-Enterobacter* group were predominantly recovered from sewage samples (67.8%) and from water samples collected downstream from STPs (55.5%), which reveals the environmental contamination by these strains. The predominance of *Klebsiella-Enterobacter* group among *bla*_KPC-or-NDM_ strains from water matrices impacted by sewage discharge has been frequently reported [18, 20, 21, 24].

*Aeromonas* spp. can carry a diverse set of chromosomal narrow-spectrum β-lactamases including the carbapenem-hydrolyzing enzyme CphA [34]. However, it is assumed that CphA does not often confer in-vitro resistance to carbapenems [35–37]. Indeed, in this study 52% of CR *Aeromonas* strains harbored *bla*_KPC_ genes. Our data also showed that sewage treatment contributes to the environmental spread of these strains. Most of the *bla*_KPC_-positive *Aeromonas* strains were recovered from TS and DW samples. Other studies have reported the occurrence of *bla*_KPC_-positive *Aeromonas* strains in TS and recreational water samples collected at downstream sites from STPs [21, 23, 38, 39]. Furthermore, *Aeromonas* strains have been recognized as an emerging cause of human waterborne infections involving recreational activities and ingestion of foods. Due to the occurrence of CphA in *Aeromonas* spp., third- and fourth-generation cephalosporins are indicated for empirical treatment of *Aeromonas* infections [36, 40, 41]. Nonetheless, the environmental spread of the *bla*_KPC_-positive isolates poses a worrying challenge to the treatment of waterborne infections thought to be produced by *Aeromonas* spp.

Resistance genes found in human pathogens are increasingly recognized in saprophytic GNB recovered from environmental matrices. Human infections caused by *K. ascorbata* are sporadically reported but include a variety of clinical presentations such as bacteremia, soft tissue infections, intra-abdominal abscesses, ventilator-associated pneumonia and urinary and biliary tract infections [3, 4, 42]. Differently, *P. agglomerans* is recognized as one of the most common saprophytic species involved in human infections, frequently resistant to cephalosporins [43]. It is of concern that broader resistance profiles have been detected in these saprophytes. Sporadic studies have already reported carbapenem-resistance genes *bla*_NDM_ and *bla*_VIM_ in clinical isolates of *P. agglomerans* [44, 45], as well as *bla*_KPC_ gene in *K. ascorbate* [3, 42]. The spread of carbapenem resistance towards unusual Enterobacterales species is an issue of concern from the “one-health” standpoint. These bacteria, although commonly regarded as avirulent, can spread resistance genes among pathogenic and commensal species as well as among patients and in the environment [42].

Some research groups argue that CR-GNB isolates carried by hospital sewage are responsible for the contamination of aquatic matrices [12–14, 18, 46]. Differently, the emergence of CR-GNB isolates in aquatic matrices is also thought to occur due to the exposure to a variety of anthropogenic pollutants, including antibiotics and heavy metals [46–48]. Additionally, aquatic matrices would provide a conducive environment for the development of antibiotic resistance even when there are no hospitals nearby [28, 47]. Our results pointed out the impact of both the community and hospital settings on the spread of *bla*_KPC-or-NDM_ strains through sewage. The frequency of *bla*_KPC-or-NDM_ strains increased as a function of the number of sewer connections, hospitals and hospital admissions, and as a function of the occurrence of district hospitals located near the STPs. Some reports acknowledge that both the hospital settings and the community are important in the dissemination of antibiotic resistance [28].

The use of antibiotics is not restricted to the clinic or hospital settings. Antibiotics are also employed in intensive livestock farms, where antibiotics are used for disease treatment of animals and for animal growth promotion [49]. The usage of antibiotics in food-producing animals selects resistant bacteria and results in the presence of antibiotic residues in farming effluents. Therefore, the environment impacted by livestock farming has been regarded as reservoirs for resistant bacteria [46]. In our study, five STP’s (STP-3, 4, 10, 12 and 14) are located in areas where intensive livestock farms exist within a 3 km radius from them. However, specifically with regard to carbapenem resistance, the occurrence of *bla*_KPC-or-NDM_ strains was not statistically associated with the presence of intensive livestock farms near STPs. Although Brazilian legislation has not completely banned antimicrobial growth promoters [50], the use β-lactans agents as animal growth promoters is prohibited in Brazil (Ministry of Agriculture, Livestock and Food Supply – Normative Ruling N° 26, July 9, 2009).

Studies worldwide have shown the resilience of CR bacteria subjected a variety of sewage treatments, including tertiary-level treatments followed by final disinfection steps [18, 21, 39, 51, 52]. In the United States, CR bacteria were recovered from 42% of STPs using chlorination for disinfection and from 12% of STPs using ultraviolet radiation [21]. In this study, 11% of the *bla*_KPC-or-NDM_ strains were recovered from TS samples from STPs which apply diverse setups of tertiary treatments, such as UNITANK (STP-4), high-rate algal pond followed by overland flow (STP-6), high-rate algal pond followed by polishing pond and chemical polishing (STP-10) and activated sludge followed by chemical polishing (STP-13).

Moving beyond hospital settings, sewage and STPs, clinically relevant *bla*_KPC-or-NDM_ CR-GNB strains have been isolated from recreational waters in other Brazilian cities such as Rio de Janeiro [23, 24, 38]. In this paper, strains of *Klebsiella* spp. *Enterobacter* spp. *Aeromonas* spp. and *Citrobacter* spp., all positive for *bla*_KPC-or-NDM_ (*n* = 25), were recovered from 6 out of 14 sites downstream from the STPs. Among those, 8 strains were recovered from superficial waters of Lake Paranoá, downstream from STP-5 and STP-13 (Fig. 2). In Brasília, a variety of spots for recreational activities, including bathing, fishing, and sailing, are located along the lakeside of Paranoá Lake. Health implications of recreational exposure to CR-GNB remain uncertain and are scarcely explored. However, the ingestion of water during recreational activities is recognized as an exposure route for asymptomatic colonization of humans [53].

Despite the variety of *bla*_KPC-or-NDM_ GNB recovered, we recognize some limitations in our study. The culture-dependent approach is prone to overlook part of the environmental microbiota, notably fastidious and underrepresented species. Therefore, our results possibly uncover only a fraction of the CR isolates carried by sewage.

## Conclusions

Clinically relevant CR-GNB species (including *Klebsiella* spp., *Enterobacter* spp. and *Aeromonas* spp.) bearing resistance genes of public health concern (*bla*_KPC_ or *bla*_NDM_) spread through sewerage and frequently prove to be resistant to sewage treatments, therefore remaining viable in the receiving water bodies. The presence of *bla*_KPC-or-NDM_ strains in sewage is statistically correlated with variables linked to the community (number of sewer connections and occurrence of nearby hospitals) and to hospital settings (number of hospitalizations). Aquatic matrices, mainly those impacted by sewage, should be subjected to surveillance of difficult-to-treat antibiotic-resistant bacteria as a strategic measure against antibiotic resistance.

## Methods

### Sample collection

Three rounds of sewage and receiving water body sampling were carried out in April, July and August 2017 covering 14 sewage treatment plants (STPs), all located in Brasília. The profile of the regions serviced by the STPs as well as the burden on those plants represented by hospital and farming activities were displayed in Table 1. A total of 138 samples were collected, including sewage samples (*n* = 70) and water samples (*n* = 68). Sewage samples were collected in STPs directly from sewage inlet pipe [Raw Sewage (RS) - *n* = 35] and directly from treated sewage outlet pipe [Treated Sewage (TS) - *n* = 35]. Water samples were collected in receiving water bodies on spots located 50 m upstream [Upstream Water (UW) – *n* = 30] and 50 m downstream [Downstream Water (DW) – *n* = 38] from the point where sewage outlet pipe discharged the treated sewage. With regard to STP-13 and STP-5, the water body receiving the treated sewage is Lake Paranoá (Fig. 2). Given the absence of appreciable water streams on the lakeside, the water samples collected from the lake were all considered downstream water samples. STP-4 and STP-10 have contiguous treatment plants occupying an area of 1.1 km^2^ and discharge their effluents in adjacent spots located at the same water body (Melchior River). In order to evaluate the role of STP-4 and STP-10 in spreading antibiotic resistance into receiving water body, their results were then pooled. Samples were collected in sterile conical tubes (50 mL) during the mornings, preserved at room temperature in insulated boxes, and sent for culture at the Central Laboratory for Public Health (LACEN-DF) in the same work shift.

### Selective culture for carbapenem-resistant (CR) gram-negative bacilli (GNB)

Five hundred microliters of each sample were cultured in Luria-Bertani broth supplemented with vancomycin [7.5 mg/L] and ertapenem [2.5 mg/L] at 36.5 °C for 24 h. Positive CR cultures were streaked on chromogenic and differential agar (ChromID® ESBL – bioMérieux) and incubated at 36.5 °C for 24 h for isolation and presumptive identification of Gram-negative bacilli (GNB). Three colonies of each presumptive bacterial group were isolated per sample limited to a maximum of 10 colonies per sample. Colonies were preserved into a semisolid nutrient medium (0.8% agar) stored in hermetically sealed tubes (3 mL) at room temperature and were kept safe from direct light exposure.

### Species identification

The bacterial isolates were recovered and grown on Mueller Hinton agar for 24 h at 37 °C for obtaining isolated colonies. The identification was accomplished using Vitek MS system (Matrix-assisted laser desorption ionization time of flight mass spectrometry - MALDI-TOF MS system - BioMerieux) in accordance with the manufacturer’s instructions. *Escherichia coli* strain ATCC™ (American Type Culture Collection) 8739 was used as a positive control. The Myla® database was accessed for the identification of bacterial isolates. A confidence level greater than 80% was adopted for genus assignment and greater than 90% for species.

### Resistance genotyping

Carbapenemase genes were detected by standard polymerase chain reactions (PCR). Supernatants derived from bacterial suspensions in deionized water and treated by boiling (100 °C for 15 min) were used as the source of DNA template. Primers were used to detect multiple alleles of carbapenemase genes (*bla*_KPC_, *bla*_NDM_, *bla*_IMP_, *bla*_VIM_ and *bla*_OXA-48_) (supporting information Table 1 and supporting information Fig. 1) [7]. Multiplex PCR was applied to detect alleles of the gene *bla*_VIM_. Additionally, primers (supporting information Table 1) were designed to specifically detect *bla*_KPC_-harboring Tn*4401* (based on the GenBank sequence CP039969.1) (supporting information Fig. 2). The forward primer (position: 83980..83999) targets the Tn*4401* transposase gene IS*Kpn6,* while the reverse primer (position: 84630..84614) recognizes the *bla*_KPC_ locus located 250-bp downstream from IS*kpn6*.

### Statistical analysis

Statistical analyses were performed using the IBM® SPSS® Statistics software (version 20). Non-parametric analyses were carried out with Fisher’s Exact Test (2-sided). Linear-by-linear associations of ordered categories were assessed with the Mantel-Haenszel test. With regard to continuous variables, non-parametric Mann-Whitney U-tests were performed to compare differences between independent groups. Results with *p* ≤ 0.05 were considered to be statistically significant.

## Supplementary Information

**Additional file 1.**

## Data Availability

The datasets generated and analyzed during the current study are available in the Figshare repository, https://figshare.com/s/67b41b9021059b41ee6d.

## Abbreviations

CR: Carbapenem-resistant
MDR: Multidrug-resistant
GNB: Gram-negative bacilli
bla: β-lactamase
STP: Sewage treatment plant
RS: Raw sewage
TS: Treated sewage
UW: Upstream water
DW: Downstream water
