# Supplementary material for: Environmental spreading of clinically relevant carbapenem-resistant gram-negative bacilli: the occurrence of blaKPC-or-NDM strains relates to local hospital activities
Source: BMC Microbiol. 2022 Jan 4;22:6. doi: 10.1186/s12866-021-02400-1 (PMC8725513; doi:10.1186/s12866-021-02400-1)
Supplement: Supplementary file 1 — Additional file 1. [file 12866_2021_2400_MOESM1_ESM.docx]

Supporting information

**Environmental spreading of clinically relevant carbapenem-resistant Gram-negative bacilli: the occurrence of *bla*_KPC-or-NDM_ strains relates to local hospital activities**

Alex Leite Pereira^a^, Pâmela Maria de Oliveira^a^, Célio Faria-Junior^b^, Everton Giovanni Alves^b^, Glaura Regina de Castro e Caldo Lima^b^, Thaís Alves da Costa Lamounier^a^, Rodrigo Haddad^a^, Wildo Navegantes de Araújo^a^

Supporting information table 1– Primers used for the resistance genotyping

| Gene (alleles) | Primer sequence 5´-3´ | Amplicon  (pb) | Tm  (ºC) | Ref. |
| --- | --- | --- | --- | --- |
| *bla_KPC_*  _(alleles 1-4; 14; 18; 21; 45)_ | (F) TGTCACTGTATCGCCGTC  (R) TCAGTGCTCTACAGAAAACC | 1010 | 58 | [5] |
| *bla*_NDM_  _(alleles 1-18)_ | (F) GGTTTGGCGATCTGGTTTTC  (R) GGCCTTGCTGTCCTTGATC | 512 | 57 | [5] |
| *bla_IMP_*  _(alleles 1-3; 5-10; 13-15; 17-20; 23-25; 28; 30; 32-34; 37; 39; 40; 42; 45; 48; 49; 52-56; 60-63; 66; 69-72; 75-80)_ | (F1) CATTTCCATAGCGACAGCAC  (F2) AACACGGTTTGGTGGTTCTT  (R) GGACTTTGGCCAAGCTTCTA | F1 - 309  F2 - 440 | 55 | [5] |
| *bla_VIM_*  _(alleles 1-20; 23-51; and 54)_ | (F) GATGGTGTTTGGTCGCATATC  (R) CTCGATGAGAGTCCTTCTAGAG | 332 | 56 | [5] |
| *bla*_OXA-48_  _(alleles 48, 162, 163, 181, 199, 204, 232, 244, 245, 247, 252, 370, 405, 416, 438, 439, 484, 505, 514, 515, 517, 538, 547)_ | (F) GCGTGGTTAAGGATGAACAC  (R) ATCATCAAGTTCAACCCAACC | 440 | 56 | [5] |
| *Tn4401*  _(alleles 1a and 1b)_ | (F) GAAGATGCCAAGGTCAATGC  (R) GGCACGGCAAATGACTA | 651 | 57 | This study |

Supporting information table 2 – Distribution of taxonomic groups and detected carbapenemase genes sorted by type of samples

|  | **SAMPLES** | | | |
| --- | --- | --- | --- | --- |
|  | **Raw Sewage**  (n = 35) [%] | **Treated Sewage**  (n = 35) [%] | **Downstream Water**  (n = 38) [%] | **Upstream Water**  (n = 30) [%] |
|  |  |  |  |  |
| **Carbapenem-resistant culture** | 35 [100]^a^ | 28 [80.0]^b^ | 27 [71.0]^c^ | 7 [23.3]^a;b;c^ |
|  |  |  |  |  |
|  | **Carbapenem-resistant isolates sorted by sample type** | | | |
|  | (n = 131) [%] | (n = 92) [%] | (n = 84) [%] | (n = 18) [%] |
| **Taxonomic groups** |  |  |  |  |
| **KE group** | 58 [44.2]^a^ | 28 [30.4]^b^ | 30 [35.7]^c^ | 0 [0]^a;b;c^ |
| *Klebsiella* spp. | 41 [31.2]^a^ | 21 [22.8]^b^ | 14 [16.6] | 0 [0]^a;b^ |
| *Enterobacter* spp. | 17 [12.9] | 7 [7.6] | 16 [19.0]* | 0 [0]* |
|  |  |  |  |  |
| ***P. putida* group** | 36 [27.4]^a^ | 31 [33.7]^b^ | 27 [32.1]^c^ | 12 [66.6]^a;b;c^ |
| ***P. aeruginosa* group** | 12 [9.1] | 3 [3.2] | 0 [0] | 0 [0] |
| **Mesophilic *Aeromonas*** | 9 [6.8] | 5 [5.4] | 7 [8.3] | 0 [0] |
| ***Pseudomonadeae*** | 6 [4.5] | 7 [7.6] | 7 [8.3] | 2 [11.1] |
| ***Citrobacter* spp.** | 4 [3.0] | 0 [0] | 2 [2.3] | 0 [0] |
| ***Stenotrophomonas maltophilia*** | 2 [1.5] | 7 [7.6] | 5 [5.9] | 1 [5.5] |
| ***Acinetobacter* spp.** | 1 [0.7] | 3 [3.2] | 0 [0] | 0 [0] |
| ***Serratia marcescens*** | 1 [0.7] | 1 [1.0] | 0 [0] | 0 [0] |
| **Enterobacterales spp.**  **(Single-occurrence isolate)** | 1 [0.7] | 0 [0] | 2 [2.3] | 0 [0] |
| ***P. fluorescens* group** | 1 [0.7] | 4 [4.3] | 4 [4.7] | 0 [0] |
| ***Chromobacterium violaceum*** | 0 [0] | 0 [0] | 0 [0] | 3 [16,6] |
| ***Proteae*** | 0 [0] | 3 [3.2] | 0 [0] | 0 [0] |
|  |  |  |  |  |
|  | **Carbapenemase genes in isolates** | | | |
|  | (n = 127) [%] | (n = 88) [%] | (n = 83) [%] | (n = 18) |
|  |  |  |  |  |
| *bla*_KPC_ | 51 [40.2]^a^ | 15 [17.0]* | 20 [24.1]^b^ | 0 [0]^a;^*^;b^ |
| *bla*_NDM_ | 16 [12.6] | 11 [12.5] | 7 [8.4] | 0 [0] |
| *bla*_OXA-48_ | 4 [3.1] | 0 [0] | 0 [0] | 0 [0] |
| *bla*_IMP_ | 2 [1.6] | 0 [0] | 4 [4.8] | 0 [0] |
|  |  |  |  |  |
|  | ***bla*_KPC-or-NDM_-positive isolates recovered from each sample** | | | |
|  | (n = 64) [%] | (n = 26) [%] | (n = 27) [%] | (n = 0) [%] |
| **Taxonomic groups** |  |  |  |  |
| **KE group** | 45 [70.3] | 17 [65.3] | 19 [55.5] | 0 [0] |
| *Klebsiella* spp. | 34 [53.1] | 16 [61.5] | 13 [48.1] | 0 [0] |
| *Enterobacter* spp. | 11 [17.2] | 1 [3.8] | 6 [22.2] | 0 [0] |
|  |  |  |  |  |
| ***Citrobacter* spp.** | 4 [6.2] | 0 [0] | 2 [7.4] | 0 [0] |
| ***P. putida* group** | 5 [7.8] | 0 [0] | 0 [0] | 0 [0] |
| ***P. aeruginosa* group** | 4 [4.7] | 1 [3.8] | 0 [0] | 0 [0] |
| **Mesophilic *Aeromonas*** | 4 [6.2] | 5 [19.2] | 4 [14.8] | 0 [0] |
| ***Pseudomonadeae*** | 2 [3.1] | 0 [0] | 0 [0] | 0 [0] |
| **Enterobacterales spp.**  **(Single-occurrence isolates)** | 1 [1.6] | 0 [0] | 2 [7.4] | 0 [0] |
| ***Proteae*** | 0 [0] | 3 [11.5] | 0 [0] | 0 [0] |
| Superscript letters indicate p < 0.05; and asterisk indicates p = 0.068 | | | | |

Supporting information table 3 - Significance levels (p*) for association of STP attendance parameters and occurrence of *bla*_KPC-or-NDM_ strains in RS samples

|  | **Analysis of Continuous Data** | **Analysis of Categorized Data** | |
| --- | --- | --- | --- |
| **STP attendance parameters** | Mann-Whitney test* | Fisher's exact test* | Mantel-Haenszel test  (Linear association) |
| Flow of treated sewage | 0.146 | 0.529 | 0.261 |
| Number of sewer connections | 0.309 | 0.029 | 0.027 |
| Total number of hospitals | 0.001 | 0.004 | 0.026 |
| Number of district hospitals | 0.004 | 0.018 | 0.013 |
| District hospital at less than 3 Km from STPs | - | 0.010 | 0.004 |
| Number of hospitalizations | 0.017 | 0.009 | 0.004 |
| Pig/poultry farming at less than 3 Km from STPs | 0.583 | 0.454 | 0.589 |
| Agricultural employment in local economy | 0.018 | 0.045 | 0.028 |
| *Two-sided test |  |  |  |

Supporting information figure 1 - Representative electrophoresis images (2% agarose gel) showing (A) *bla*_NDM_ (512 bp) and (B) *bla*_KPC_ (1010 pb) amplicons. Arrangement of the lanes in both gels: lane 1, *K. pneumoniae* strain IOC-4955 positive for *bla*_KPC_; lane 2, *K. pneumoniae* strain ATCC-700603 (negative control strain); lane 3, *E. cloacae* strain CCBH-10892 positive for *bla*_NDM_. Non-duplicated sewage isolates were showed in lanes 4-12. Ladder indicates 100 bp DNA molecular marker with reference bands at 600, 1,500 and 2,000 bp.

Supporting information figure 2 - Design of Tn*4401* primers. (A) Physical map of Tn*4401* harboured by the plasmid p1701_01 (GenBank sequence CP039969.1:82000-87000) in *bla*_KPC_-positive *K. pneumoniae* strain R1701. Black arrows delimit the IS*Kpn6*-*bla*_KPC_ intergenic sequence (651 bp) targeted by Tn*4401* primers. (B) Representative electrophoresis images (2% agarose gel) showing Tn*4401* amplicons (651 bp). Ladder indicates 100-bp DNA molecular marker displaying reference bands at 600, 1,500 and 2,000 bp. Lanes 1-8, *bla*_KPC_-positive isolates; lane 9, *K. pneumoniae* IOC-4955 control strain for *bla*_KPC_; lane 10, *K. pneumoniae* reference strain ATCC-700603 (*bla*_KPC_ -negative strain).


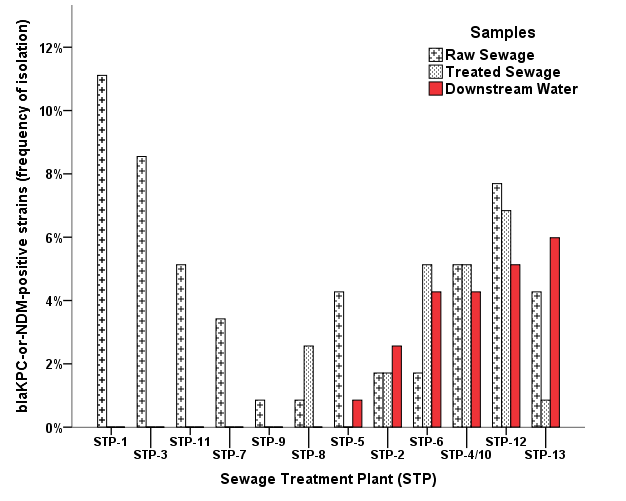


Supporting information figure 3 **– *bla*_KPC-or-NDM_ strains resist the sewage treatment and remain viable in downstream sites of the receiving water bodies.** Notice that STP-4 and STP-10 (STP-4/10) discharge their treated effluents in the same receiving water body, and for this reason their data were pooled for the downstream site analyses.
